# Tumor-specific CD4^+^ T cells develop cytotoxic activity and eliminate virus-induced tumor cells in the absence of regulatory T cells

**DOI:** 10.1007/s00262-012-1329-y

**Published:** 2012-08-14

**Authors:** Ilseyar Akhmetzyanova, Gennadiy Zelinskyy, Simone Schimmer, Sven Brandau, Petra Altenhoff, Tim Sparwasser, Ulf Dittmer

**Affiliations:** 1 Institute for Virology, University of Duisburg-Essen, Virchowstr 179, 45147 Essen, Germany; 2 Department of Otorhinolaryngology, University Clinics Essen, 45122 Essen, Germany; 3 Institute for Infection Immunology, TWINCORE, Feodor-Lynen-Str. 7, 30625 Hannover, Germany

**Keywords:** Tumor immunity, Viral oncogenesis, Effector CD4^+^ T cells, Regulatory T cells

## Abstract

The important role of tumor-specific cytotoxic CD8^+^ T cells is well defined in the immune control of the tumors, but the role of effector CD4^+^ T cells is poorly understood. In the current research, we have used a murine retrovirus-induced tumor cell line of C57BL/6 mouse origin, namely FBL-3 cells, as a model to study basic mechanisms of immunological control and escape during tumor formation. This study shows that tumor-specific CD4^+^ T cells are able to protect against virus-induced tumor cells. We show here that there is an expansion of tumor-specific CD4^+^ T cells producing cytokines and cytotoxic molecule granzyme B (GzmB) in the early phase of tumor growth. Importantly, we demonstrate that in vivo depletion of regulatory T cells (Tregs) and CD8^+^ T cells in FBL-3-bearing DEREG transgenic mice augments IL-2 and GzmB production by CD4^+^ T cells and increases FV-specific CD4^+^ T-cell effector and cytotoxic responses leading to the complete tumor regression. Therefore, the capacity to reject tumor acquired by tumor-reactive CD4^+^ T cells largely depends on the direct suppressive activity of Tregs. We suggest that a cytotoxic CD4^+^ T-cell immune response may be induced to enhance resistance against oncovirus-associated tumors.

**Electronic supplementary material:**

The online version of this article (doi:10.1007/s00262-012-1329-y) contains supplementary material, which is available to authorized users.

## Introduction

The majority of tumor viruses are well controlled by the immune system and therefore cause only transient or no disease in their hosts after infection. It is apparent that the main role in this control can be ascribed to the presence of cytotoxic CD8^+^ T cells, which are very effective in destroying virus infected or transformed cells. In recent years, the idea that CD4^+^ T cells can also play a considerable role in protective anti-tumor responses has received growing attention. One important function of CD4^+^ T cells is their help for CD8^+^ T cell and antibody responses against virus or tumor antigens. However, studies using several tumor models have shown that CD4^+^ T cells can efficiently eliminate major histocompatibility complex (MHC) class II expressing tumor cells [1, 2] as well as tumors lacking MHC class II molecules [3–5], demonstrating a direct role of CD4^+^ T cells in tumor rejection. However, whether CD4^+^ T cells can fully compensate for the effector functions of CD8^+^ T cells in the control of virus-induced tumors remains unclear. In addition, the significance of regulatory T cells (Tregs) in inhibiting tumor-specific CD4^+^ T-cell responses during tumor rejection in vivo has not been defined. It was previously reported that Tregs infiltrate tumors and draining lymph nodes, suggesting that they interfere with anti-tumor immune responses in general and thereby contribute to tumor growth and progression [6]. Tregs inhibit the function of many adaptive and innate immune cells, including CD4^+^ T cells, CD8^+^ T cells, dendritic cells (DCs), macrophages, natural killer (NK) cells, and B cells through various molecular mechanisms [7]. To define the role of Tregs in tumor immunity, most studies have used malignant tumor models, in which the immune system failed to prevent tumor progression. However, the task of effector CD4^+^ T cells and Tregs should also be analyzed in effective anti-tumor immunity to fully understand their role in tumor biology.

To address these questions, we have used the Friend retrovirus (FV)-induced mouse tumor cell line of C57BL/6 origin, called FBL-3 cells [8]. This cell line was generated by inoculation of FV complex into mice, which results in an EPO receptor-dependent proliferation signal in erythroid precursor cells. Integration of pro-virus can subsequently cause over-expression of the *Spi1* proto-oncogene and inactivation of the *p53* tumor suppressor gene resulting in host cell transformation. Thus, FV infection can induce fully malignant erythroleukemia in susceptible mouse strains. FBL-3 is a FV-transformed tumor cell line that does not produce infectious virus, but expresses highly immunogenic FV antigens [9, 10]. After subcutaneous (s.c.) implantation of FBL-3 cells into mice, the tumor grows locally and subsequently regresses in a CD8^+^ T-cell-dependent manner over a time period of 20 days [11, 12]. Tumor-specific CD4^+^ T cells seem to be less important for tumor rejection when functional CD8^+^ T cells are present [13]. However, if the pool of Tregs is expanded by a chronic infection, mice fail to reject transplants of FBL-3 tumors due to a Treg-mediated suppression of tumor-specific CD8^+^ T-cell responses [11]. In the present study, we used Foxp3 (forkhead box P3) transgenic mice expressing the diphtheria toxin (DT) receptor under the control of the Foxp3 promoter, which made it possible to selectively deplete Tregs in vivo and to determine the influence of Foxp3^+^ Tregs on T-cell responses during tumor regression. We especially focused on the direct anti-tumor effect of CD4^+^ T cells and found that these cells could fully compensate for the lack of cytotoxic CD8^+^ T cells when their functional suppression by Tregs was interrupted.

## Materials and methods

### Mice

Experiments were done using sex- and age-matched C57BL/6 (B6), CD45.1, and DEREG [14] mice that were between 8 and 10 weeks old when experiments started. Mice were housed in specific pathogen-free conditions and treated in accordance with institutional guidelines.

### Cell lines

FBL-3 is an FV-induced tumor cell line derived from a C57BL/6 mouse [8]. The highly immunogenic FBL-3 cell line expresses FV antigens but does not produce infectious virus. FBL-3 cells were maintained in complete RPMI medium supplemented with 10 % FCS and 0.5 % penicillin/streptomycin.

### Tumor challenge

1 × 10^7^ FBL-3 tumor cells were injected s.c. on the right flank in 100 μl of PBS through a 27-gauge needle on day 0. In order to verify tumor volume by external caliper, the greatest longitudinal diameter (length) and the greatest transverse diameter (width) were determined. Tumor size based on caliper measurements was calculated by the formula: tumor area (cm^2^) = π × *a* × *b*, where *a* = half of length and *b* = half of width. After 4, 6, 8, 11, 15, and 20 days, mice were killed, and tumors and draining and non-draining lymph nodes were resected.

### In vivo cell depletion

CD8 depletion was performed as described [15] and started at day 0 and carried out every other day for the tumor growth analysis until mice were killed due to the progressive tumor growth, and four times (on days 0, 2, 4, 6) for the experiments where mice were killed at day 6 post-tumor inoculation. Depletion of Tregs was done as described [16] and started at day 1 for three times. Depletion of CD4^+^ T cells was performed as described [17] and started at day 0. Deletion of NK cells was carried out as described [15].

### Staining and flow cytometry

Antibodies used for cell-surface staining were anti-CD4 (AF 700-conjugated, GK 1.5), anti-CD8a (eFluor 450-conjugated, 53–6.7), anti-CD43 (PerCP-conjugated, 1B11), anti-CD25 (PE Cy7-conjugated, PC61.5), anti-Mac-1 (anti-CD11b) (FITC-conjugated, WT.5), anti-F4/80 (PE-conjugated, BM8), anti CD86 (eFluor 605-conjugated, GL-1), and Fc block anti-mouse CD16/CD32 (93) (eBioscience). Dead cells were excluded by using propidium iodide. Intracellular granzyme B (GzmB) staining was performed as described [18]. To determine intracellular production of interferon-γ (IFN-γ), tumor necrosis factor-α (TNF-α) and interleukin-2 (IL-2) cells from lymph nodes were stimulated in the presence of 2 μg/ml of CD28 antibody and 2 μg/ml of brefeldin A for 5 h at 37 °C. The cells were then stained for surface expression of CD4, CD8, and CD43, fixed, and permeabilized with Cytofix/Cytoperm solution (BD). The cells were then washed, permeabilized, and incubated with Fc blocking anti-mouse CD16/CD32. After that, cells were labelled with monoclonal antibodies specific for IL-2, IFN-γ, TNF-α, and anti-CD154. In addition to cytokines, cells were labeled with anti-CD154 (PE-conjugated, CD40Ligand, gp39, MR1). Foxp3 expression was detected by intracellular staining using an anti-mouse/rat Foxp3 antibody (FITC-conjugated, FJK-16s) and the Foxp3 staining kit (eBioscience). Helios expression was measured by intracellular staining using an anti-mouse/human Helios antibody (eFluor 450-conjugated, 22F6, BioLegend) and the Foxp3 staining kit (eBioscience). Dead cells were excluded by using Fixable Viability Dye (eBioscience). Data were acquired on an LSRII flow cytometer (Becton–Dickinson) from 200,000 to 500,000 lymphocyte-gated events per sample. Analyses were done using FACSDiva software (Becton–Dickinson) and FlowJo software (Treestar).

### In vivo cytotoxicity

The in vivo cytotoxicity assay was done as it was described by Barber et al. [19]. Tumor-bearing DEREG mice were depleted or not depleted for their Treg and CD8^+^ T cells. Six days after tumor challenge, all groups of mice received Carboxyfluorescein succinimidyl ester (CFSE)-labeled lymphocyte targets loaded with peptide [20] as well as unloaded unstained cells from CD45.1 mice as a control population. 2 h after intravenous (i.v.) injection of donor cells, mice were killed and in vivo killing activity was quantified in single-cell suspensions from the drLN of each tumor-bearing mouse.

### Immunohistochemistry

Tumors were dissected sharply using surgical scissors, immediately shock-frozen in liquid nitrogen and stored at −80 °C. The frozen tissues were sectioned in 5 μm slices, placed on slides, air-dried, and stained with hematoxylin–eosin.

Frozen samples were air-dried and fixed with Cytofix/Cytoperm (BD, Heidelberg, Germany). Endogenous peroxidase activity was blocked with Dako REAL Peroxidase-Blocking solution (DakoCytomation, Hamburg, Germany) followed by several washing steps with PBS. Slides were incubated for 60 min with the primary monoclonal rat anti-mouse antibodies anti-CD11b antibody (BioSource, Solingen, Germany) or anti-CD4 antibody (BD Bioscience, Heidelberg, Germany). Subsequently, samples were incubated with peroxidase-conjugated rabbit anti-rat (Dianova, Hamburg, Germany) and goat anti-rabbit antibodies (Dianova) for 30 min each and AEC Single Solution (Invitrogen). Nuclei were visualized by Shandon Instant Hematoxylin (Thermo Fisher Scientific). Sections were analyzed with a Zeiss Axioscope 2 (Carl Zeiss, Oberkochen, Germany) using objective lenses with 200× magnification and AxioVision software (Zeiss).

### Tetramer staining

MHC class I and class II tetramer staining was performed as described [17].

### Statistical analyses

Statistical analyses and graphical presentations were computed with Graph Pad Prism version 5. Statistical differences (*P* value) between two groups were calculated using unpaired t test. Statistical differences (*P* value) between the different parameters were calculated testing with the Kruskal–Wallis one-way analysis of variance on ranks and Newman–Keuls multiple comparison tests.

## Results

### Kinetics of the antigen-specific CD4^+^ and CD8^+^ T-cell response in lymph nodes during tumor rejection

**Fig. 1:**
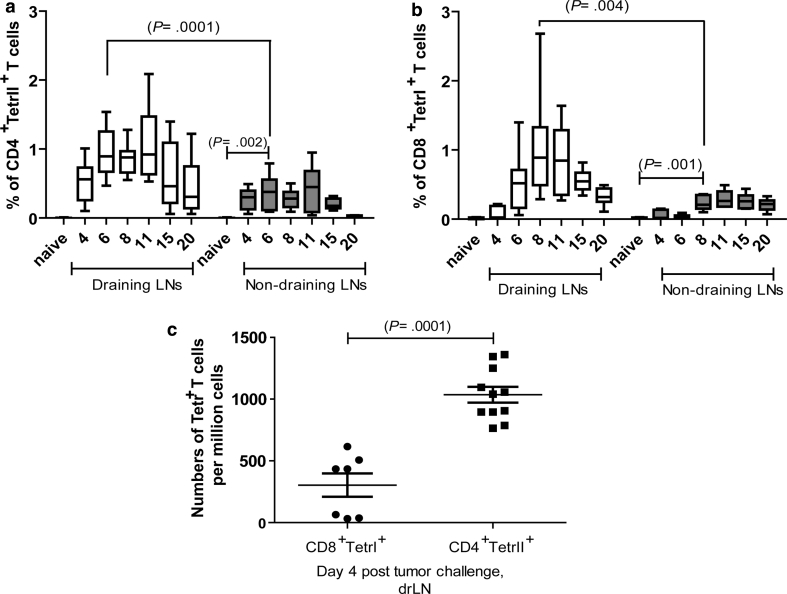
Kinetics of FBL-3-specific effector CD4^+^ and CD8^+^ T-cell responses: B6 mice were inoculated s.c. with 1 × 10^7^ FBL-3 cells (*n* = 9–12 mice per group). Mean percentages ± SEM of FBL-3-specific CD4^+^TetII^+^ T cells reactive with I-A^b^ MHC class II tetramers specific for FV-Env epitope (**a**) and effector CD8^+^ T cells reactive with MHC class I H-2D^b^ tetramers specific for the FV gagL CTL epitope (**b**) in draining (*white box plots*) and non-draining (*grey box plots*) lymph nodes. The mean percentage for each group is indicated by a *line*. **c** Expansion of antigen-specific CD4 T cells in draining lymph nodes at day 4 ptc is shown. Each *dot* represents an individual mouse, and the mean numbers are indicated by a *line*. All tetramer-positive T cells expressed cell-surface activation marker CD43. Statistically significant differences between the groups are given in the figures. The experiment was repeated three times with comparable results

To study T-cell responses in tumor cell rejection, we used the leukemia cell line FBL-3, a FV-induced tumor line from a C57Bl/6 mouse. These highly immunogenic murine leukemia cells induce local tumor growth after s.c. injection into C57/Bl6 mice for about 20 days before being rejected due to IFN-γ and granzyme-producing CD8^+^ T cells [11]. It has been shown that FBL-3 tumor cells express FV antigens that can be recognized by CD8^+^ and CD4^+^ T cells [9, 10]. To determine the kinetics of T-cell responses in this tumor rejection model, we quantified the population of FV-specific effector CD8^+^ T cells by staining lymphocytes from draining (drLN) and non-draining lymph nodes (non-drLN) of FBL-3-challenged mice with H-2D^b^gagL MHC class I tetramers [9, 20] or MHC class II tetramers loaded with the H-2I-A^b^-restricted CD4^+^ T-cell epitope H19-Env [20]. Early after tumor challenge (4 days post-tumor challenge (ptc)), expansion of specific cells was only found in the CD4^+^ but not the CD8^+^ T-cell population (Fig. 1a, b). Thus, the frequencies of antigen-specific CD4^+^ T cells in drLN at day 4 ptc were significantly higher compared to specific CD8^+^ T cells (Fig. 1c). Peak expansion of specific CD4^+^ T cells was found as early as at 6 days post-tumor challenge, whereas CD8^+^ T-cell expansion reached its maximum 2 days later (Fig. 1a, b). For both T-cell populations, the contraction phase began at day 15 ptc. A comparison between different lymph nodes showed that the specific CD4^+^ and CD8^+^ T-cell responses were generally located in drLN as the peak expansion of T cells was significantly higher in drLN than in non-drLN. However, a modest increase in the percentage of specific CD4^+^ and CD8^+^ T cells was also observed in non-drLN compared to lymph nodes cells from naïve animals (Fig. 1a, b). Collectively, the data demonstrate a local expansion of tumor-specific T cells with the CD4^+^ T-cell response developing more rapidly than the CD8^+^ T-cell response.

### Functional activity of the tumor-specific CD4^+^ and CD8^+^ T cells

**Fig. 2:**
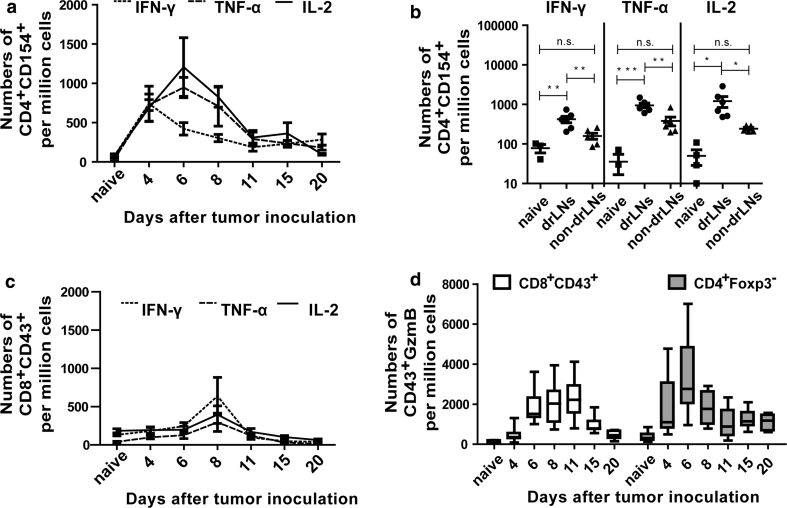
Cytokines and functional properties of T cells: B6 mice were inoculated s.c. with 1 × 10^7^ FBL-3 cells. At different time points ptc, lymphocytes from lymph nodes were isolated and investigated. Kinetics of IFN-γ-, TNF-α-, and IL-2 expressing CD154^+^CD4^+^ (**a**) and CD43^+^CD8^+^ (**c**) T cells from lymph nodes are shown. **b** Numbers of cytokine producing CD4^+^CD154^+^ T cells at day 6 ptc are depicted. Each *dot* represents an individual mouse and the means are indicated by a *line*. **d** Intracellular expression of GzmB. Numbers of CD8^+^CD43^+^ (*white box plots*) and CD4^+^Foxp3^−^ (*grey box plots*) T cells producing GzmB are shown in drLNs at different days ptc. Differences between two groups are indicated (*P < 0.05, **P < 0.005, ***P < 0.0005). All experiments were repeated three times with comparable results

Next, we wanted to analyze functional properties of CD4^+^ and CD8^+^ T cells during tumor rejection. To this end, we performed kinetic analysis of cytokine and granzyme B (GzmB) expression in T cells after tumor challenge. To analyze the total populations of CD4^+^ and CD8^+^ T cells that were activated during tumor rejection, we used the maker CD154 (CD40L) for CD4^+^ T cells [21] and the activation-associated glycoform of CD43 for CD8^+^ T cells [22]. In order to exclude Tregs from the effector CD4^+^ T-cell pool, we also stained for intracellular expression of Foxp3. In drLN nodes, the highest frequency of CD4^+^ T cells producing the three cytokines IFN-γ, TNF-α, and IL-2 was found between day 4 and 6 ptc (Fig. 2a). At day 8 ptc, the cytokine response already started to decrease, which was earlier than the decline in tetramer II-positive CD4^+^ T cells (Figs. 1a, 2b). The frequency of CD4^+^CD154^+^ T cells producing the three cytokines was significantly higher in drLN than in non-drLN (Fig. 2b and supplementary figure S1, available on-line), which correlated with the increased percentages of tetramer-positive CD4^+^ T cells in drLN (Fig. 1a). The peak cytokine production by CD8^+^ T cells in drLN was found at day 8 ptc again showing the delay in the CD8^+^ T-cell response compared to CD4^+^ T cells (Fig. 2a, c). In addition, the numbers of CD8^+^CD43^+^ T cells producing cytokines were much lower than those of activated CD4^+^ T cells. No cytokine production by CD8^+^ T cells was found in non-drLN during tumor rejection (data not shown). As expected, the cytotoxic molecule GzmB was also produced by CD8^+^CD43^+^ T cells after tumor challenge (Fig. 2d). The peak of this functional response was found between day 8 and 11 ptc. Remarkably, tumor-induced activation of Foxp3^−^ CD4^+^ T cells in drLN also resulted in their differentiation into GzmB-producing cells, suggesting a potential cytotoxic role for these cells. Peak GzmB expression in CD4^+^CD43^+^ T cells was observed at day 6 ptc, which was again earlier as in the CD8^+^ T-cell compartment. Thus, both CD8^+^ and CD4^+^ T-cell populations in drLN expressed pro-inflammatory cytokines and GzmB in response to tumor challenge, but the CD4^+^ T-cell response initiated earlier and the magnitude of the response was higher than the CD8^+^ T-cell response.

### Kinetics of the local Treg response during tumor rejection

**Fig. 3:**
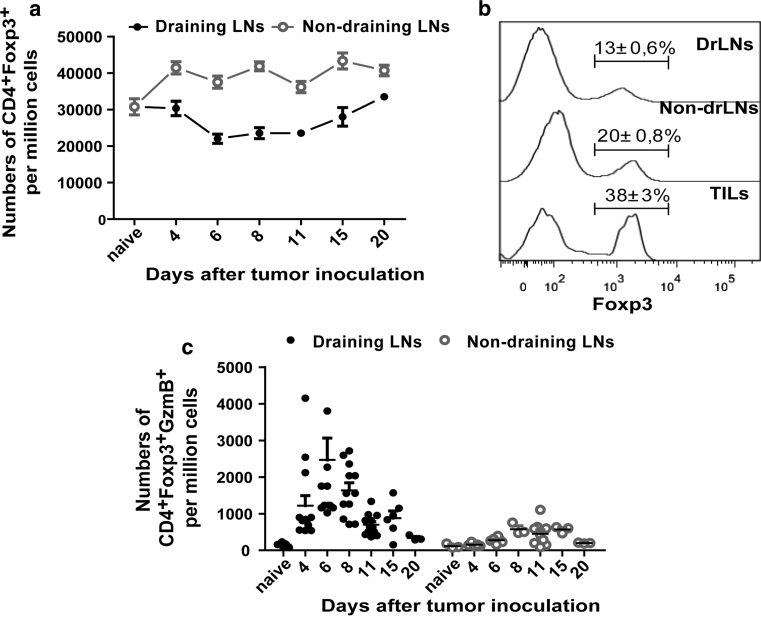
Regulatory T cells in lymph nodes: B6 mice were inoculated s.c. with 1 × 10^7^ FBL-3 cells on day 0. **a** Numbers of CD4^+^Foxp3^+^ in drLNs and non-drLNs are shown. **b** Representative histograms display Foxp3 expression among CD4^+^ T cells in lymph nodes and tumor at day 6 ptc [Tumor-infiltrating lymphocytes (TILs)]. Numbers indicate percentages within the respective Foxp3^+^ gate. **c** Numbers of CD4^+^Foxp3^+^ T cells producing GzmB in lymph nodes are shown. Each *dot* represents an individual mouse, and the mean numbers are indicated by a *line*. The experiment was repeated three times with comparable results

Since Tregs were reported to have a suppressive role in the control of local tumor immune responses [6], we next assessed the significance of these cells in rejection of the FBL-3 tumor cells. To this end, the kinetics of Treg responses in drLN and non-drLN were compared. To distinguish between Treg and effector CD4^+^ T-cell populations, we utilized the unique Treg marker Foxp3. Interestingly, in non-drLN, the frequency of Foxp3^+^ Tregs started to increase at day 4 ptc and stayed elevated until day 20 ptc in comparison with naïve animals (Fig. 3a). In contrast, in drLN Treg, frequencies decreased on day 4 ptc and remained reduced until day 15 ptc. This was a surprising finding since we knew from previous studies in the FV model that Tregs expand at the side of inflammation during a chronic virus infection [16]. One possible explanation was that these cells leave the drLN and migrate into the tumor microenvironment. To address this, tumor-infiltrating lymphocytes (TIL) were isolated and examined for Treg frequencies. From the TIL, a mean of 38 % Tregs was found at 6 days ptc, whereas only a mean of 13 % Tregs was found in drLN, suggesting an infiltration of Tregs from the drLN into the tumor microenvironment.

One of the most potent mechanisms of Treg-mediated effector T-cell suppression is the direct killing of effector cells by the granzyme/perforin pathway [23, 24]. Thus, we examined the production of GzmB in CD4^+^Foxp3^+^ Tregs. In drLN, Tregs started to produce GzmB at 4–6 days ptc (Fig. 3c) and the frequency of GzmB-producing Tregs correlated with the kinetics of the overall CD4^+^ T-cell response (Fig. 1a). In contrast, in non-drLN, FBL-3 challenge did result in only slight expansion of GzmB-producing Tregs at day 8–15 ptc (Fig. 3c). To analyze whether the GzmB^+^ Tregs were natural (nTregs) or induced Tregs (iTregs), we stained for the nTreg marker Helios, a member of the Ikaros transcription factor family [25, 26]. The vast majority of the granzyme-producing Tregs expressed Helios (Supplementary figure S2, available on-line), suggesting that those cells were mostly thymic-derived nTregs.

### The role of different T-cell populations in the control of tumor growth

**Fig. 4:**
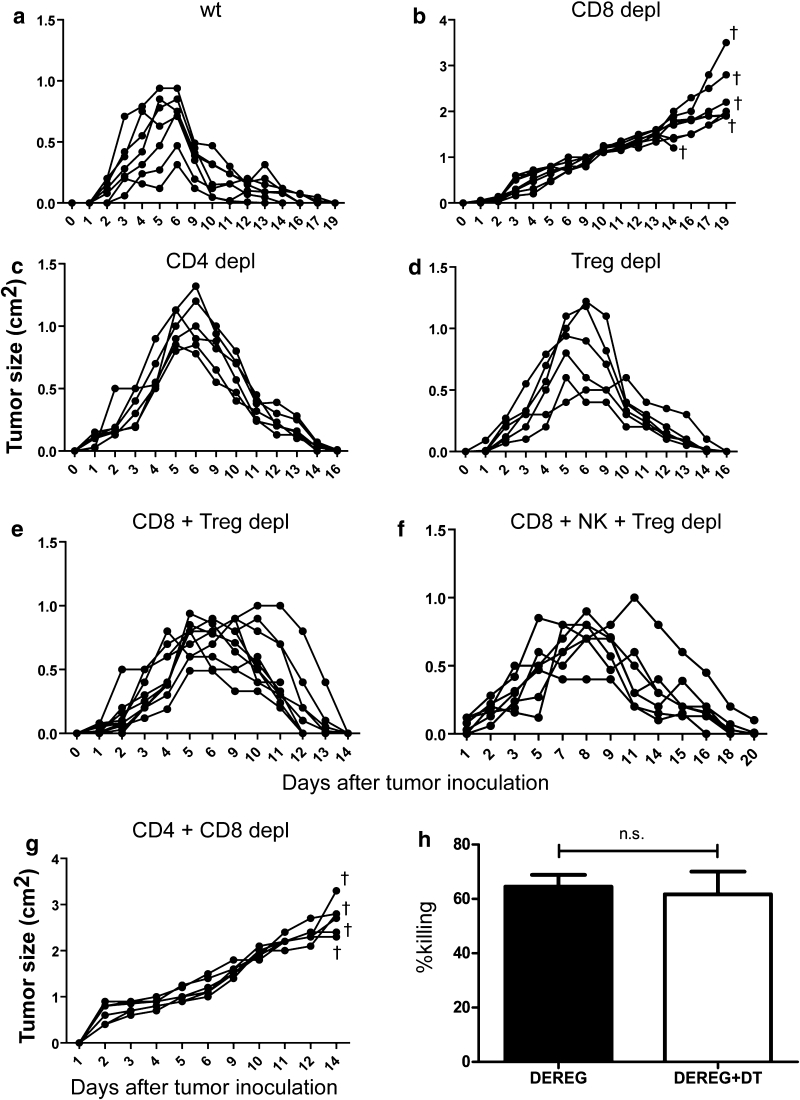
Influence of different cell populations on tumor formation: Effects of no depletion (injected with PBS) (**a**), depletion of CD8^+^ T cells (**b**), CD4^+^ T cells (**c**), Tregs (**d**), CD8^+^ and Tregs (**e**), CD8^+^, NK cells and Tregs (**f**) and CD4^+^ and CD8^+^ T cells (**g**) are shown. DEREG mice were injected s.c. with 1 × 10^7^ FBL-3 cells (1 × 10^7^) and tumor size was measured. Mice were depleted for their CD8^+^, CD4^+^ T cells, and Tregs as described in “Materials and methods.” Each *line* represents tumor progression in an individual mouse. **h** In vivo killing activity of CD8^+^ T cells in different treatment of mice. Mean percentages show killing of cells loaded with the FV D^b^gagL peptide in an in vivo CTL assay (described in “Materials and methods” section) at day 6 ptc in DEREG mice treated or not treated with DT. Data were pooled from four to six independent experiments with similar results. *P* values were determined by an unpaired *t* test (*n.s*., non-significant). *Dragger symbol* mice were euthanized due to progressive tumor growth

It was previously reported that CD8^+^ T cells are essential in controlling FBL-3 progression, whereas CD4^+^ T cells did not affect the tumor growth [11, 13]. In agreement with these previous studies, tumor regression was completely abrogated when CD8^+^ T cells were ablated by monoclonal antibodies (Fig. 4b). In contrast, the depletion of CD4^+^ T cells did only temporary increase the tumor size at 6 days ptc but did not affect the subsequent rejection of FBL-3 tumor cells (Fig. 4c). The data demonstrate that mainly CD8^+^ T cell-mediated rejection of FBL-3 tumor cells but CD4^+^ T cells had only a minor effect. However, the influence of Tregs on the different effector T-cell populations during tumor rejection is poorly understood. To investigate this influence, we studied tumor regression and T-cell functions after selective depletion of Tregs. We used transgenic DEREG mice, which express a diphtheria toxin (DT) receptor under control of the *Foxp3* promoter. An injection of DT selectively depleted more than 90 % of the Tregs (data not shown). No other cell population was depleted by this treatment. Interestingly, DT treatment of tumor-bearing DEREG mice did not significantly improve tumor elimination (Fig. 4d). Thus, in a tumor model in which immune surveillance and immune control are effectively mediated by tumor-specific CD8^+^ T cells, these cells do not seem to be functionally suppressed by Tregs. This is in line with our finding that depletion of Tregs did not enhance FBL-3-specific cytotoxicity of CD8^+^ T cells in an in vivo CTL assay with target cells loaded with a Friend virus immunodominant epitope peptide [9] (Fig. 4h). However, these results did not indicate whether or not tumor-specific CD4^+^ T-cell responses were suppressed by Tregs. To clarify this, we depleted Tregs and CD8^+^ T cells at the same time in tumor-bearing mice. Whereas CD8^+^ T-cell ablation alone resulted in progressive tumor growth (Fig. 4b), additional Treg depletion enabled the mice to again reject the tumor (Fig. 4e). The only difference to non-depleted wild-type mice was that the FBL-3 tumors increased in size for up to 4 days longer before they were rapidly rejected. To exclude a possible role of NK cells, the additional depletion of those cells in the group of mice lacking Tregs and CD8^+^ T cells was performed (Fig. 4f). Such mice could still reject the tumor, demonstrating that NK cells had no effect on tumor rejection in CD8^+^-depleted mice. In order to demonstrate that tumor rejection in mice depleted for CD8^+^ T cells and Tregs (Fig. 4e) was due to effector CD4^+^ T-cell responses, tumor growth was tested in mice lacking both CD4^+^ (including Tregs) and CD8^+^ T cells. In the absence of these T-cell compartments, no control of tumor growth was observed (Fig. 4g). This experiment suggests that CD4^+^ T cells can mediate potent anti-tumor effects when cytotoxic CD8^+^ T cells are absent but that they are tightly controlled in their activity by Foxp3^+^ Tregs.

### The impact of Tregs on the functions of tumor-specific CD4^+^ T cells

**Fig. 5:**
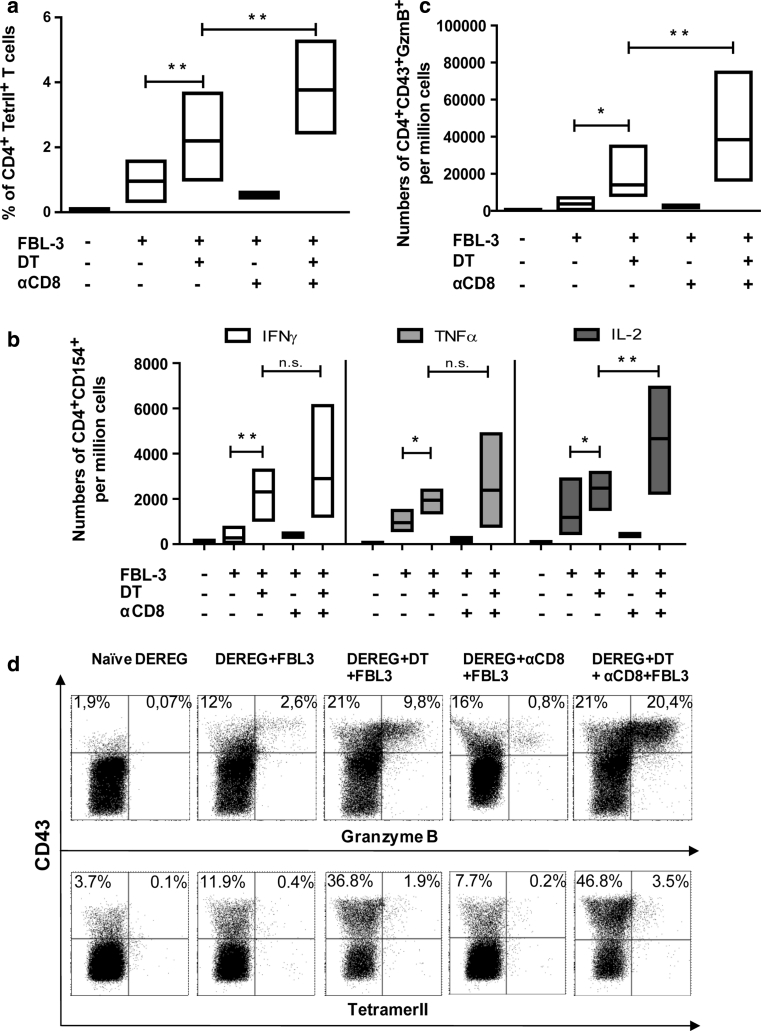
The influence of regulatory T cells on tumor-specific CD4^+^ T-cell functions: DEREG mice were inoculated s.c. with 1 × 10^7^ FBL-3 cells on day 0. One day before tumor inoculation, some mice also received DT to deplete Foxp3^+^ Tregs, and day later monoclonal antibody to deplete CD8^+^ T cells. At day 6 post-tumor transplantation, lymphocytes from draining lymph nodes were analyzed. **a** The percentages of CD4^+^ T cells reactive with I-A^b^ MHC class II tetramers are shown. Numbers of CD4^+^CD154^+^ T cells producing cytokines (IFN-γ, TNF-α, and IL-2) are shown (**b**). Numbers of activated (positive for the activation-induced isoform CD43) CD4^+^Foxp3^−^ T cells producing GzmB (**c**) and representative *dot* plots of GzmB and tetramer II expression (**d**) in different treatment of mice are shown. Differences between two groups are indicated (*P < 0.05, **P < 0.005). Results were obtained from three experiments with comparable results

To prove that Tregs indeed control anti-tumor CD4^+^ T-cell functions in the FBL-3 model, we analyzed numbers of tumor-specific CD4^+^ T cells, their cytokine production and cytotoxic activity after Treg ablation with or without additional CD8^+^ T-cell depletion. In DT-treated DEREG mice challenged with tumor cells for 6 days, we observed a significant increase in the mean percentage of tumor-specific (tetramer II^+^) CD4^+^ T cells in comparison with mice that received only FBL-3 cells (Fig. 5a). Moreover, if depletion of Tregs was combined with CD8^+^ T-cell removal, the CD4^+^ T-cell response was further significantly enhanced. CD8^+^ T-cell depletion alone did not influence the mean percentage of tumor-specific CD4^+^ T cells, suggesting that their expansion was mainly controlled by Tregs (Fig. 5a). Tregs did not only influence CD4^+^ T-cell expansion but also modified their functional properties. In DT-treated mice, significantly more CD4^+^CD154^+^ T cells expressed the cytokines IFN-γ, TNF-α, and IL-2 than in mice receiving only tumor cells (Fig. 5b). Dual depletion of Tregs and CD8^+^ T cells resulted in slightly higher mean frequencies of cytokine producing CD4^+^ T cells than after Treg deletion alone but this difference was only significant for IL-2-producing cells (Fig. 5b). To determine possible cytotoxic effects against FBL-3 tumor cells, production of the cytolytic molecule GzmB by activated (CD43^+^) CD4^+^ T cells was analyzed. In mice lacking Tregs, the frequency of GzmB-positive cells was significantly higher compared to non-depleted tumor-bearing mice (Fig. 5c, d). Additional ablation of CD8^+^ T cells together with the Tregs resulted in a significant rise in the mean frequencies of GzmB^+^ CD4^+^ T cells in comparison with mice only depleted for Tregs (Fig. 5c). Again, CD8^+^ T-cell depletion alone did not influence the functional CD4^+^ T-cell response during tumor rejection (Fig. 5c, d).

### The cytotoxic potential of tumor-specific CD4^+^ T cells in vivo

**Fig. 6:**
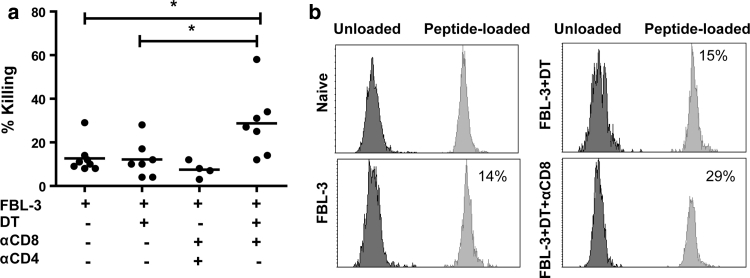
In vivo killing activity of CD4^+^ T cells after different treatment of mice: Mean percentages of killing (**a**) and representative histograms (**b**) of in vivo CTL assay are depicted. Tumor-bearing mice were depleted for their Tregs alone or additionally for their CD8^+^ T cells. Target cells from donor CD45.1 mice (CFSE^+^ and CFSE^−^) were co-transferred i.v. in the same amount into tumor-bearing mice. CFSE^+^ cells were loaded with the class II-restricted peptide recognized by CD4^+^ T cells, whereas CFSE^−^ cells were used as a control population 2 h later, lymphocytes were isolated from the draining lymph nodes and analyzed by flow cytometry to determine the percentage of remaining target cells that are either CFSE^+^ or CFSE^−^. Each *dot* represents an individual mouse, and the mean percentages are indicated by a *line*. Differences between two groups are indicated (*P < 0.05). All experiments were repeated two times with comparable results

To analyze whether the increased expression of GzmB correlated with improved tumor-specific lysis of target cells after Treg depletion, we performed a series of in vivo killing experiments. The in vivo killing activity was quantified in the drLN of each mouse during tumor rejection. In non-depleted animals, CD4^+^ T cells showed a modest in vivo killing activity not exceeding a mean of 13 % target cell lysis (Fig. 6a). Surprisingly, depletion of Tregs alone did not significantly improve the lysis of target cells. In contrast, simultaneous ablation of CD8^+^ T cells and Treg significantly enhances the killing of peptide-loaded cells (Fig. 6a, b), which correlated with the high frequency of GzmB-producing cells in this group of mice (Fig. 5c, d). Notably, in this group, tumor growth was completely rejected even in the absence of CD8^+^ T cells (Fig. 4e). To demonstrate that cytotoxic CD4^+^ T cells mediated the target cell killing in the group of CD8^+^ T cell plus Treg depleted mice, we additionally depleted the effector CD4^+^ T cells. This completely abrogated the MHC II-restricted killing activity (Fig. 6a). Collectively, these data suggest that CD4^+^ T cells can gain cytotoxic activity against tumor cells when CD8^+^ T cells are not active but this activity is tightly controlled by Tregs during tumor rejection.

### Upregulation of MHC class II molecule on FBL-3 cells in vivo

The level of MHC II expression directly influences T-lymphocyte activation and recognition of target cells by CD4^+^ T cells. It has been published previously that CD4^+^ T cells cannot directly recognize FBL-3, since they only express MHC class I but no MHC class II molecules [27]. However, our in vivo experiments strongly suggest a cytotoxic activity of CD4^+^ T cells against FBL-3 tumors. Therefore, the MHC class II expression of FBL-3 tumor cells extracted directly from tumor-bearing mice was analyzed. As expected, no MHC class II molecules were found on FBL-3 cells from cell culture (Supplementary figure S3a, available on-line). To study MHC class II expression in vivo and to distinguish the FBL-3 from tumor-infiltrating cells, CD45.1 transgenic mice were used. In these mice, all leukocytes express the CD45.1 alloantigen and therefore can be excluded by the surface staining. Interestingly, after being in the host environment, the phenotype of the FBL-3 cells had partially changed. A proportion of 14 % of the inoculated MHC II-deficient FBL-3 cells became I-A^b^-positive after 4 days ptc in CD45.1 mice (Supplementary figure S3b, available on-line). These MHC-II-expressing FBL-3 cells could be a possible target for cytotoxic CD4^+^ T cells and might explain part of the CD4^+^ T-cell-mediated FBL-3 tumor rejection (Fig. 4e).

### The role of macrophages in FBL-3 tumor growth

**Fig. 7:**
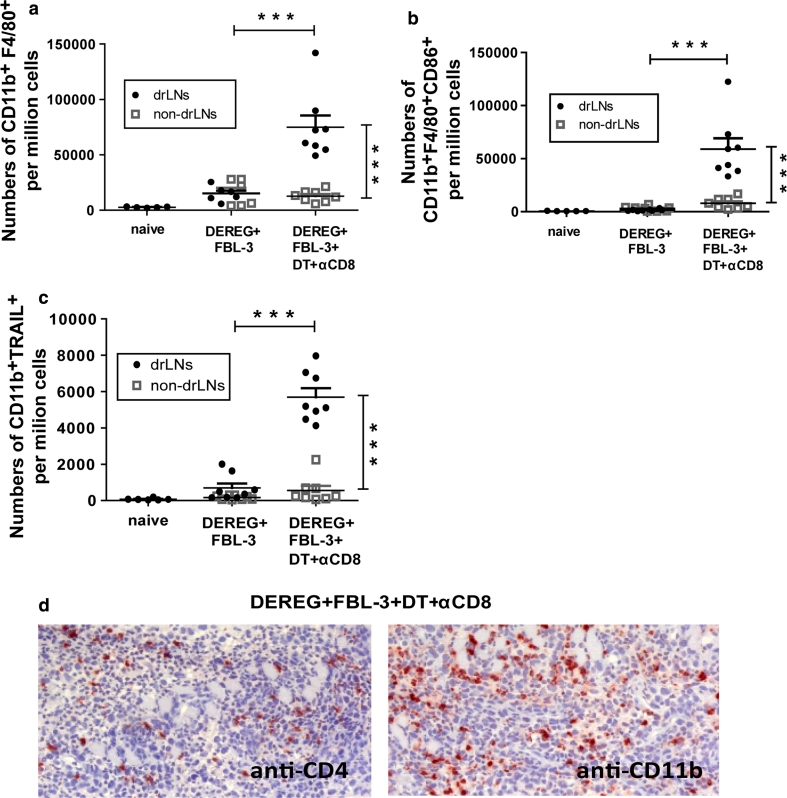
The role of macrophages in FBL-3 tumor growth: DEREG mice were inoculated s.c. with 1 × 10^7^ FBL-3 cells on day 0. One day before tumor inoculation, some mice also received DT to deplete Foxp3^+^ Tregs and day later monoclonal antibody to deplete CD8^+^ T cells. At day 6 post-tumor transplantation, leukocytes from drLNs and non-drLNs were analyzed. Numbers of CD11b^+^F4/80^+^ cells are shown (**a**). Numbers of CD11b^+^F4/80^+^CD86^+^ (**b**) and CD11b^+^TRAIL^+^ (**c**) cells are shown. Experiments were repeated twice with similar results. Differences between two groups are indicated (***P < 0.0005). The infiltration of CD4^+^ T cells and CD11b^+^ macrophages into the tumor is shown by immunohistology (**d**) (magnification ×200)

It is well known that macrophages can contribute to tumor rejection. We therefore focused our study on the role of these cells in FBL-3 tumor growth. Since tumor-bearing mice depleted for CD8^+^ T cells and Tregs showed complete tumor rejection, we analyzed in this group for CD11b^+^F4/80^+^ macrophage activation (expression of CD86 [28]) at day 6 ptc in lymph nodes in comparison with non-depleted and naïve animal. Significant expansion of CD11b^+^F4/80^+^ macrophages from drLNs but not in non-drLNs was observed (Fig. 7a). Depletion of CD8^+^ T cells and Tregs also promoted the upregulation of the costimulatory molecule CD86, which indicated macrophage activation (Fig. 7b). Interestingly, activated macrophages did not produce granzyme B (data not shown). However, significantly increased expression of the TNF-related apoptosis-inducing ligand (TRAIL) that can induce tumor cells apoptosis [29] was detected on macrophages from drLNs of mice depleted for CD8^+^ T cells and Tregs compared to non-depleted controls (Fig. 7c). These data were in line with the immunohistochemistry of tumors from the dual depleted group that showed infiltration of CD11b^+^ macrophages in the tumor mass in addition to CD4^+^ T-cell infiltration (Fig. 7d).

## Discussion

The CD4^+^-helper T-cell response represents a critical part of a functional immune system and is well characterized in many tumors but its effector role in the control of virus-induced tumors remains unclear. In the current research, we have used a highly immunogenic FV-induced tumor cell line of C57BL/6 mouse origin, namely FBL-3 cells, as a model to study the mechanisms of immunological control and escape during tumor formation. Our studies show that when CD8^+^ T cells were unable to control FBL-3 tumor development, cytotoxic effector CD4^+^ T cells were able to take over and eliminate the tumor. However, direct anti-tumor effects of CD4^+^ T cells were strictly regulated by Tregs, which thereby contributed toward tumor progression.

Here, we compared the kinetics of the CD4^+^ T-cell response to FBL-3 tumor antigen in drLN with the CD8^+^ T-cell response and found a more rapid development of the CD4^+^ T-cell response after FBL-3 injection. Peak expansion of tumor-specific CD4^+^ T cells was observed 2 days later as the CD8^+^ T cell. In contrast, in a virus infection model with FV peak tetramer-positive CD8 T-cell, responses were far greater in magnitude [18, 30] than peak tetramer-positive CD4 T-cell responses [17]. In previous studies using the Moloney murine sarcoma and leukemia virus complex (MoMSV), which share the immunodominant CD4^+^ and CD8^+^ T-cell epitopes with FV, the burst size of the virus-specific CD8^+^ T-cell response was considerably larger than that of virus-specific CD4^+^ T cells in drLNs [20].

Immune escape of tumors has been studied in many models and has in part been attributed to active suppression mediated by CD4^+^Foxp3^+^ Tregs [31]. The drLN is the site of the critical decision between immune activation and tolerance and has a major influence on host immunity. The current study shows that the Tregs from the drLNs appear to migrate into the tumor and influence the local microenvironment. The influence of Treg localization (sentinel lymph nodes and tumor) on cancer progression is still disputed [32–35]. The assessment of circulating Tregs or Tregs infiltrating the tumor itself has been used as a prognostic indicator in human cancers but conflicting results were obtained in different tumor diseases. Curiel et al. [32] reported that the presence of high numbers of CD4^+^CD25^+^Foxp3^+^ cells in malignant ascites of ovarian carcinoma correlated with tumor staging and reduced survival. In colorectal cancer patients, it was demonstrated that Tregs in drLNs correlated better with disease progression and tumor stage [33]. Another observation in breast carcinoma indicated that Treg localized within lymphoid aggregates, but not in the tumor area, had a negative impact on patients’ survival [34]. The finding agrees with animal models of colon cancer [36] and goes in line with our study. This suggests that Tregs in the drLNs can inhibit the development of effector cells such as cytotoxic CD4^+^ and CD8^+^ T cells, whereas Tregs in the tumor itself may have dual functions of controlling tumor-promoting inflammation on one hand but suppressing local effector T-cell responses on the other [37]. Thus, in studies aimed at analyzing the impact of Tregs on tumor progression, it is critical to take the localization of Foxp3^+^ Treg into account.

Although the perforin/granzyme pathway was previously known to be utilized by CD8^+^ T cells and NK cells to lyse target cells, recent studies have suggested that Tregs may also use perforin/granzyme-mediated cytotoxicity as a mechanism to kill conventional T cells [23, 24]. In our study, we showed that after FBL-3 challenge, Tregs in the drLNs started to produce GzmB. Moreover, the frequency of GzmB-producing Tregs correlated with the kinetics of the effector CD4^+^ T-cell response. This observation and data from Treg depletion experiments strongly suggest that GzmB-producing Tregs suppress anti-tumor CD4^+^ T-cell responses. Grossman et al. [38] showed that activated human Tregs expressed granzyme A and/or B and could kill activated CD4^+^ and CD8^+^ T cells. This Treg-mediated killing was perforin-dependent [38]. Similarly, Gondek et al. [23] reported that murine Tregs stimulated with anti-CD3 mAb expressed GzmB and could suppress conventional CD4^+^ T cells in a cell contact and GzmB-dependent manner. Using a system of tumor inoculation and adoptive transfer of Tregs, it was shown that GzmB was highly induced in tumor-associated Tregs by local factors in the tumor microenvironment [39]. Thus, cytotoxic activity of Tregs might be a general mechanism of Treg-mediated suppression in tumor development. Interestingly, this is different to Treg activity in chronic infectious diseases in which most activated Tregs do not express granzymes [16].

It was previously reported that CD8^+^ T cells are essential in controlling FBL-3 progression [11, 12]. After administration of anti-CD8 mAb, rejection of local tumors induced by FBL-3 challenge was blocked and mice died from systemic lymph node metastases [12]. In agreement with these previous studies, we showed that the absence of CD4^+^ T cells during FBL-3 formation did not significantly influence the tumor growth [13]. Although we found a very potent and rapid CD4^+^ T-cell response (production of cytokines and cytotoxins), these cells had only a minor effect on FBL-3 tumor rejection. Most likely, a direct anti-tumor effect of CD4^+^ T cells is not required in a system in which the cytotoxic function of CD8^+^ T cells is sufficient to mediate the successful rejection of the tumor. However, effector CD4^+^ T cells could replace cytotoxic CD8^+^ T cells but they were under tight regulation by Tregs. While CD8^+^ T-cell ablation alone resulted in uncontrolled tumor growth [12, 13], additional Treg depletion enabled the CD4^+^ T cells to reject the tumor. Control experiments showed that this anti-tumor effect was exclusively mediated by effector CD4^+^ T cells (Fig. 4g). To define the suppressive effects of Tregs on conventional T cells, one has to take their functions on CD4 and CD8 populations into account. Our current study shows that Treg depletion had no biological effect on CD8^+^ T-cell-mediated tumor rejection. Although CD8^+^ T-cell functions were augmented after Treg depletion (data not shown), this did not result in faster tumor rejection, most likely because the antitumor CTL were very efficient even under the suppressing influence of Tregs. In contrast, in FV infection, the functional suppression by Tregs mainly targeted CD8^+^ T cells [40], resulting in the development of functional exhausted CD8^+^ T cells and in high FV loads in lymphatic organs [16]. Earlier Iwashiro et al. [11] demonstrated that mice persistently infected with FV have approximately twice the normal percentage of splenic CD4^+^CD25^+^ Tregs and lose their ability to reject the implantation of FBL-3 cells. In a mouse model of spontaneous mammary carcinoma, the depletion of Tregs resulted in CD4^+^ T-cell activation and subsequent development of efficient CD8^+^ T-cell activity [41]. In two other models of antitumor immunity, it was proposed that Tregs diminished CD8^+^ T-cell function by consuming IL-2 [42] or by preventing cytotoxic granule release [43]. In a model of autoimmunity, other authors proposed that DCs were central to Treg inhibition in vivo and attenuated priming of CD4-helper cells [44]. Hence, it seems to depend on the environment and the type of immunity whether Tregs preferentially regulate CD4^+^ or CD8^+^ T-cell populations. In addition, the specificity of Treg depletion might also influence the results in certain experiments. The use of non-specific Treg targeting agents such as CD25-depleting antibodies, which in addition to CD25^+^ Tregs also deplete recently activated CD25^+^ effector T cells, can complicate the interpretation of the data. In our study, we used transgenic DEREG mice, which express a diphtheria toxin receptor under the control of the *Foxp3* promoter, allowing highly selective depletion of Foxp3^+^Tregs even during ongoing immune responses [45]. Our finding that Tregs preferentially influence the anti-tumor CD4^+^ T-cell immune response revealed several aspects that had not been reported previously. The CD4^+^ T cells residing in the drLNs of mice depleted of Tregs and CD8^+^ T cells showed major changes to those of non-depleted mice. We demonstrated that in vivo depletion of Tregs and CD8^+^ T cells in FBL-3-bearing DEREG transgenic mice augments GzmB production by CD4^+^ T cells and increases FV-specific CD4^+^ T-cell effector and cytotoxic responses leading to the complete tumor regression. Therefore, the Foxp3^+^ Tregs control the proliferation and function of effector CD4^+^ T cells and prevent the induction of efficient CD4^+^ T cells with cytotoxic potential. It was previously shown that in the murine mammary carcinoma model, deletion of Tregs resulted in increasing numbers of IFN-γ and IL-2-producing CD4^+^ T cells at tumor sites [41]. However, the CD4^+^ T cells alone, even in the absence of Treg suppression, were not sufficient to abrogate tumor progression [41] but the Treg removal was performed by using anti-CD25 antibodies and depletion of Tregs was incomplete [41]. Another group has also used DEREG mice for selective depletion of Tregs [46]. However, the regression of the melanoma growth, which was induced by depletion of Foxp3^+^ Tregs, was critically dependent on the presence of CD8^+^ T cells in this model and additional elimination of those cells resulted in ongoing tumor progression [46].

We show here that the immune system may still be capable of controlling tumor development in the absence of the main cytotoxic population (CD8^+^ T cells). However, underlying mechanisms that influence the activity of effector CD4^+^ T cells in the absence of CD8^+^ T cells are poorly defined. CD8^+^Foxp3^+^ T cells were reported to mediate immunosuppression in cancer patients [47]. Depletion of these CD8^+^Foxp3^+^ Tregs in addition to CD4^+^Foxp3^+^ Tregs might allow for antitumor cytotoxic function of CD4^+^ T cells and tumor rejection. Moreover, some of the molecules that are produced by CD8^+^ T cells, such as granzymes, may have immunomodulatory effects on other T cells [48].

To demonstrate that the increased expression of GzmB by effector CD4^+^ T cells resulted in antigen-specific cytotoxicity in vivo after depletion of Foxp3^+^ Tregs, we performed an in vivo CTL assay. CD4^+^ T cells showed potent killing activity of peptide-loaded targets in the drLN of FBL-3-bearing mice. The acquisition of cytotoxic activity by tumor-reactive CD4^+^ T cells is particularly striking since it only emerged when the suppressive function of Tregs was blocked. From previous work, we knew that CD4^+^ T cells can help rejection of tumors through indirect effects on NK cells [49] and tumor-infiltrating macrophages [1, 50, 51] and Tregs can negatively influence this help by inhibiting IFN-γ synthesis [52]. Cytotoxic CD4^+^ targeting viral antigens [53–55] and alloantigens [56–58] have been described previously, but in these models, the influence of Tregs on effector CD4^+^ T-cell activity was not investigated but the tumor or the pathogen was eliminated by adoptive transfer of effector CD4^+^ T cells [57, 59]. Quezada et al. [57] showed effector CD4^+^ T-cell-dependent tumor rejection when radiotherapy and adoptive transfer of tumor-reactive CD4^+^ T cells were combined with the blockade of the CTLA-4 molecule that is expressed on Tregs [60, 61], suggesting that Tregs influenced cytotoxic CD4^+^ T cells in this model as well.

As published previously, CD4^+^ T cells cannot directly recognize FBL-3, since they only express MHC class I but not MHC class II molecules in vitro [27] (Supplementary figure S3a,). However, our in vivo experiments strongly suggest cytotoxic activity of CD4^+^ T cells against FBL-3 tumor cells. In the current study (Supplementary figure S3b), we showed that after being in the host environment, a proportion of the inoculated MHC II-deficient FBL-3 cells became I-A^b^-positive. In addition, CD4^+^ T cells can support killing of tumor cells by indirect mechanisms including recruitment of antigen-presenting cells [27, 62] or killing stroma cells [63] that support tumor growth. A combination of such mechanisms can elucidate the antigen-specific elimination of FBL-3 tumors by cytotoxic CD4^+^ T cells.

It is well established that solid tumors are often infiltrated by macrophages. As previously published, these cells play an important role in the FBL-3 tumor model [27]. In our study, we illustrate significant expansion of activated CD11b^+^F4/80^+^ macrophages that produce TRAIL in drLNs after depletion of Tregs and CD8^+^ T cells. Moreover, infiltration of CD11b^+^ cells into the tumor mass was shown, which suggests an antitumor effector function of macrophages. Activated macrophages have been reported to secrete a number of molecules with tumorocidal effects, like nitric oxide and reactive oxygen intermediates, and thus can partly mediate antitumor activity [64]. In addition, antitumor function of macrophages can be stimulated by IFN-γ-producing CD4^+^ T cells. However, macrophages alone could not control FBL-3 cell growth in the absence of CD4^+^ and CD8^+^ T cells (Fig. 4g). Thus, we believe that tumor-specific CD4^+^ T cells can facilitate direct anti-tumor activity, which can be supported by TRAIL^+^ macrophages inducing the classical macrophage activation pathway that contributes to inhibition of tumor cell growth [65].

The direct antitumor role of CD4^+^ T cells has been controversially discussed since most tumors do not express MHC II and thus cannot be directly recognized by CD4^+^ T cells. Nonetheless, there are a number of HLA-DR-expressing tumors in patients [66]. Moreover, HLA-DR expression is associated with better prognosis in colorectal cancers [67]. The ability of MHC II-negative tumors to start to express class II molecules in the tumor microenvironment can be applied in cancer immunotherapy. Recent investigations in melanoma patients showed effectiveness of adoptive CD4^+^ T-cell therapy [68]. Thus, analyzing for HLA-DR expression of the primary tumor and metastasis and subsequent adoptive CD4^+^ T-cell transfer might be a promising approach for future immunotherapy.

Cytotoxic CD4^+^ T-cell activity against MHC II-negative tumors can also be used in cancer immunotherapy. Genetic modification of cytotoxic T cells with chimeric antigen receptors (CARs) specific for tumor antigen allows MHC-independent antigen recognition that nonetheless retains the T-cell effector mechanisms that are needed to eliminate tumor cells [69].

In conclusion, our studies have established a critical role for cytotoxic CD4^+^ T cells in the context of oncoviral diseases. We propose that effector CD4^+^ T cells, which are largely regulated by Foxp3^+^ Tregs during tumor formation, are capable of maintaining immune control against FBL-3 tumor via direct killing and can functionally replace CD8^+^ T cells. We suggest that cytotoxic CD4^+^ T-cell immune responses may be induced therapeutically to enhance resistance against oncovirus-associated tumors.

## Electronic supplementary material

Below is the link to the electronic supplementary material.

Supplementary material 1 (PDF 1321 kb)
