# Supplementary material for: Tumor-specific CD4+ T cells develop cytotoxic activity and eliminate virus-induced tumor cells in the absence of regulatory T cells
Source: Cancer Immunol Immunother. 2012 Aug 14;62(2):257–71. doi: 10.1007/s00262-012-1329-y (PMC3569596; doi:10.1007/s00262-012-1329-y)
Supplement: Supplementary file 1 — Supplementary material 1 (PDF 1321 kb) [file 262_2012_1329_MOESM1_ESM.pdf]

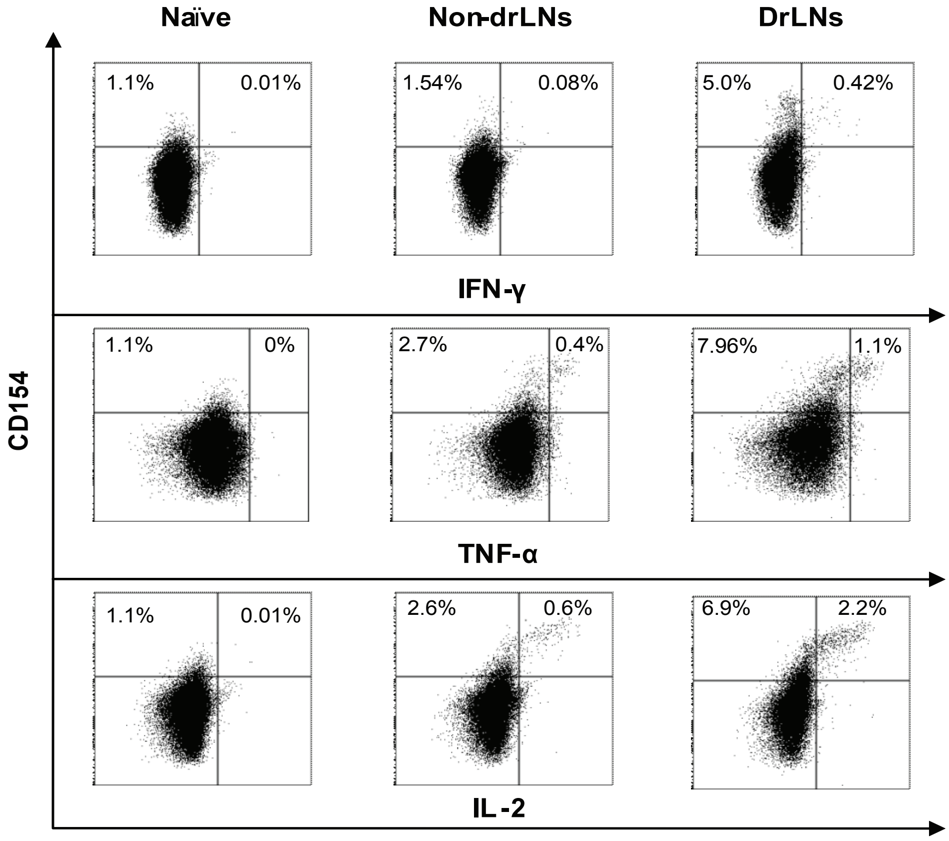

Supplementary figure S1 Cytokines producing by CD4^{+} T cells B6 mice were inoculated s.c. with $1{\times}10^{7}$ FBL-3 cells. At different time points ptc lymphocytes from lymh nodes were isolated and investigated. Representative dot plots of cytokine producing CD4^{+}CD154^{+} at day 6 ptc in lymph nodes are shown.

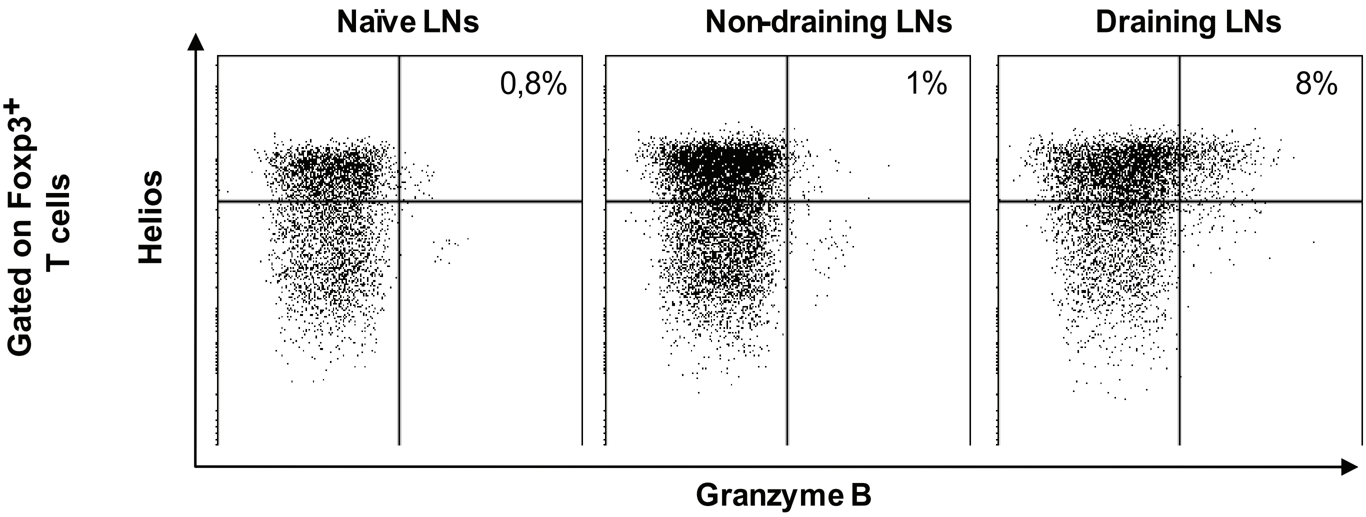

Supplementary figure S2 Granzyme B producing Tregs in lymph nodes B6 mice were inoculated s.c. with 1×10^7 FBL-3 cells on day 0. Representative FACS analysis of GzmB and Helios expression by $Foxp3^{+}CD4^{+}$ T cells in lymph nodes at day 6 post tumor inoculation is shown.

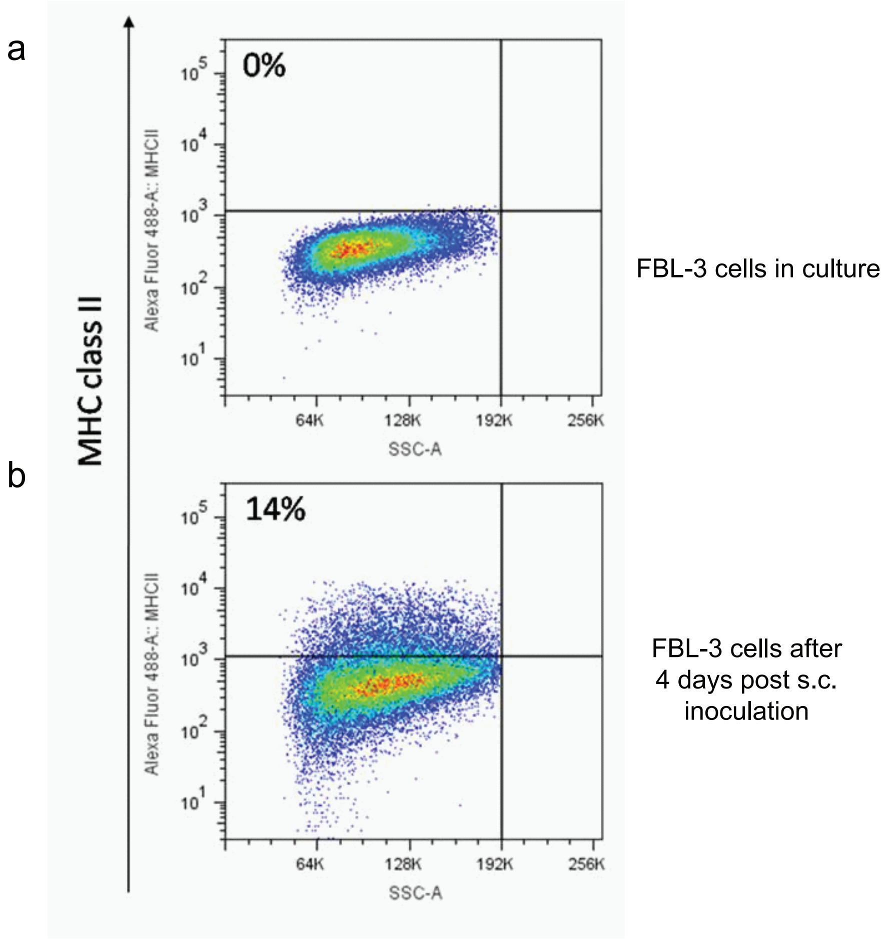

Supplementary figure S3 Expression of MHC class II molecules on FBL-3 cells Representative dot plots on MHC class II expression on FBL-3 cells. (a) Staining of FBL-3 cells from *in vitro* cell cultures (in RPMI medium). (b) FBL-3 cells taken directly *ex vivo* from the tumor of CD45.1 tumor-bearing mice. CD45.1 mice were challenged s.c. with 1×10^7 FBL-3 cells on day 0. On day 4 ptc mice were sacrificed and tumors were extracted. Expression of MHC class II molecule on the CD45.1 negative cell population (FBL-3 cells) was determined.
